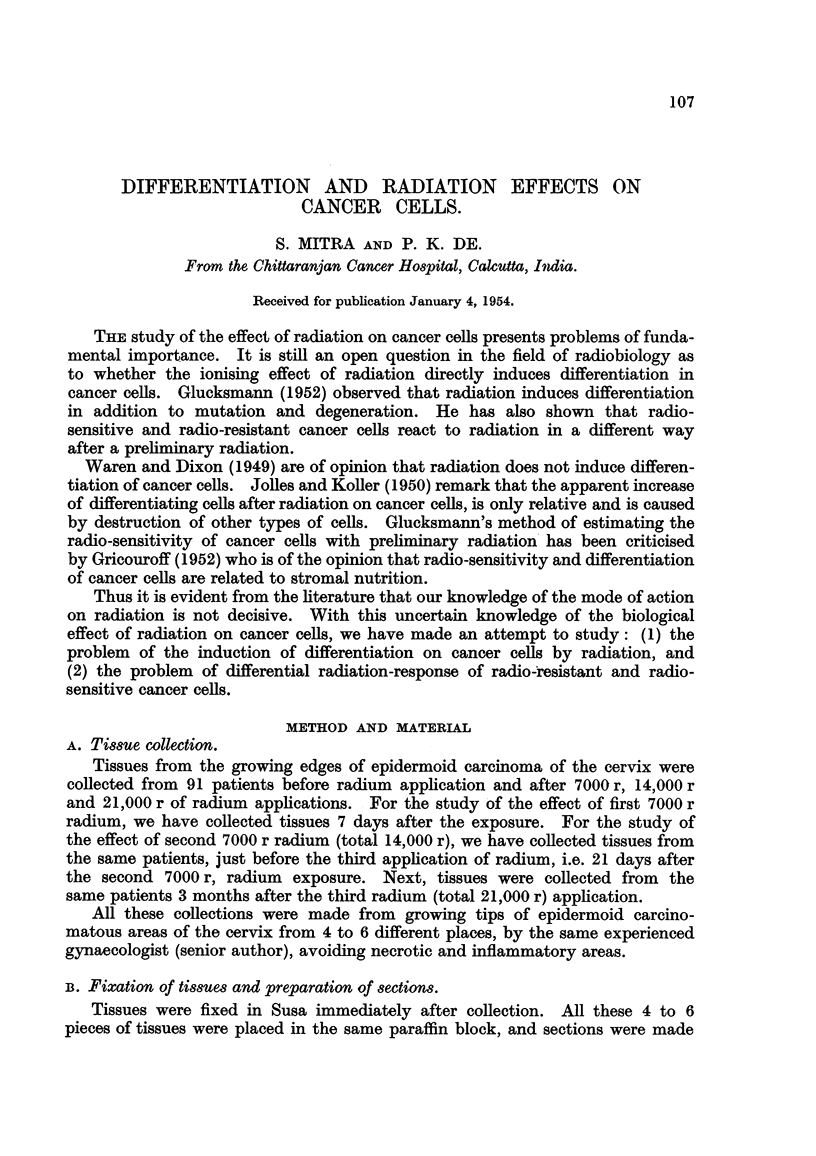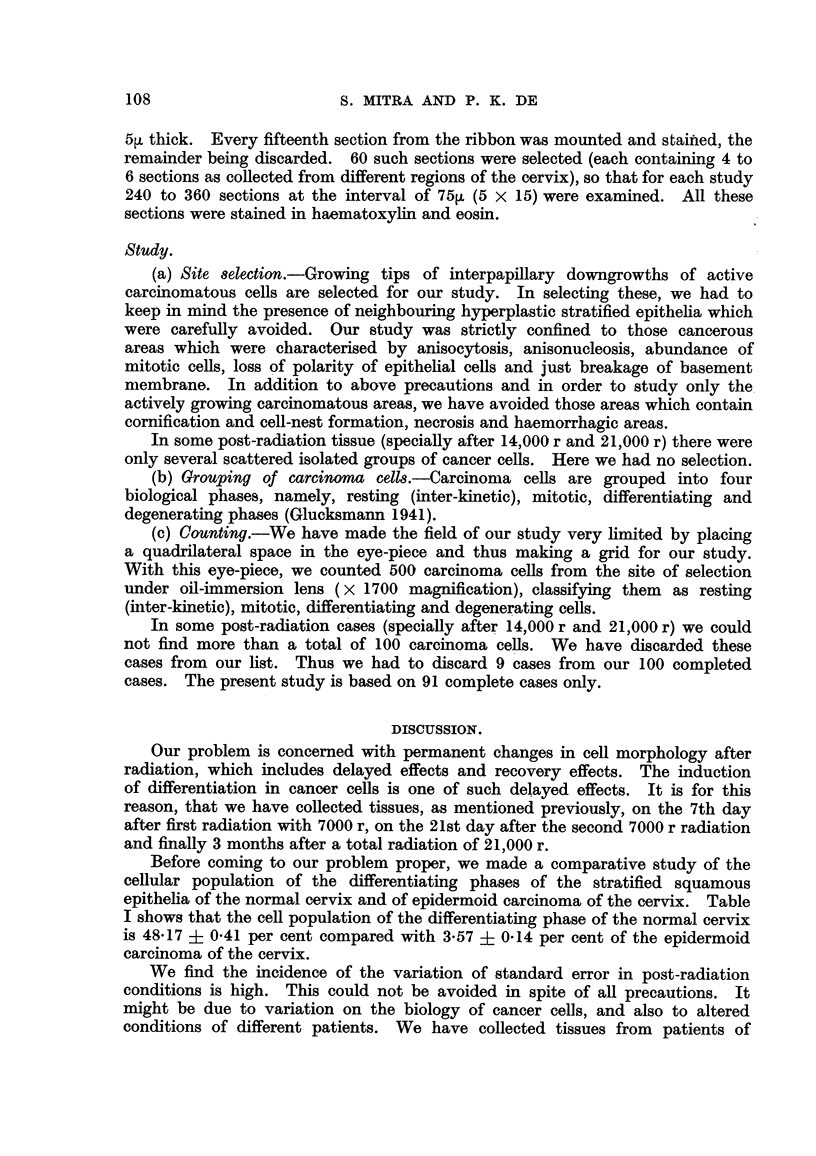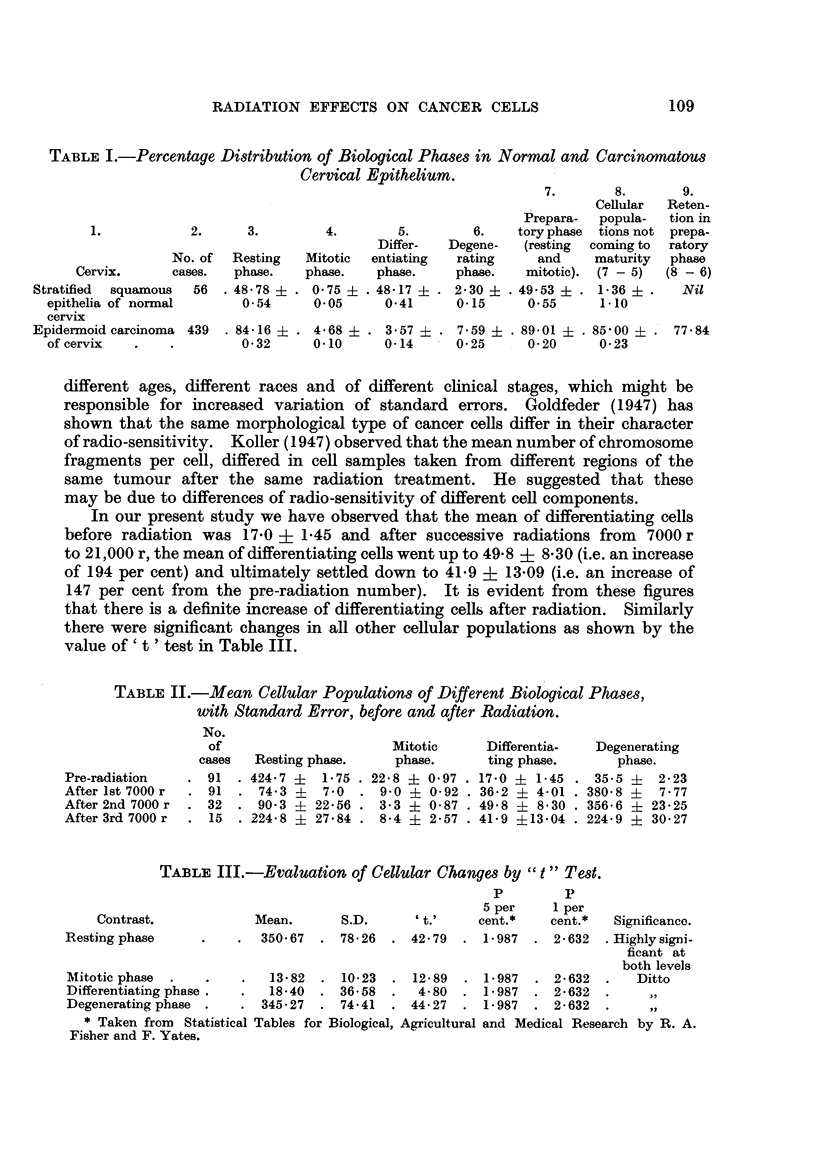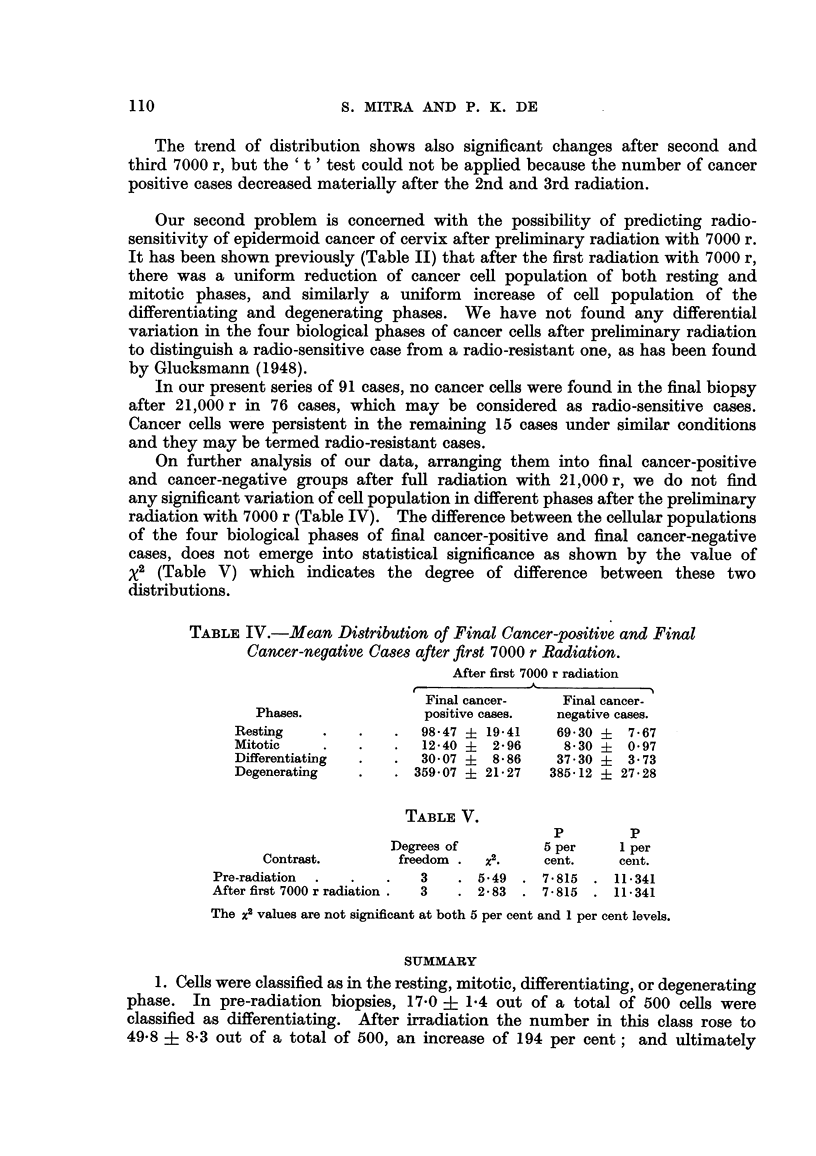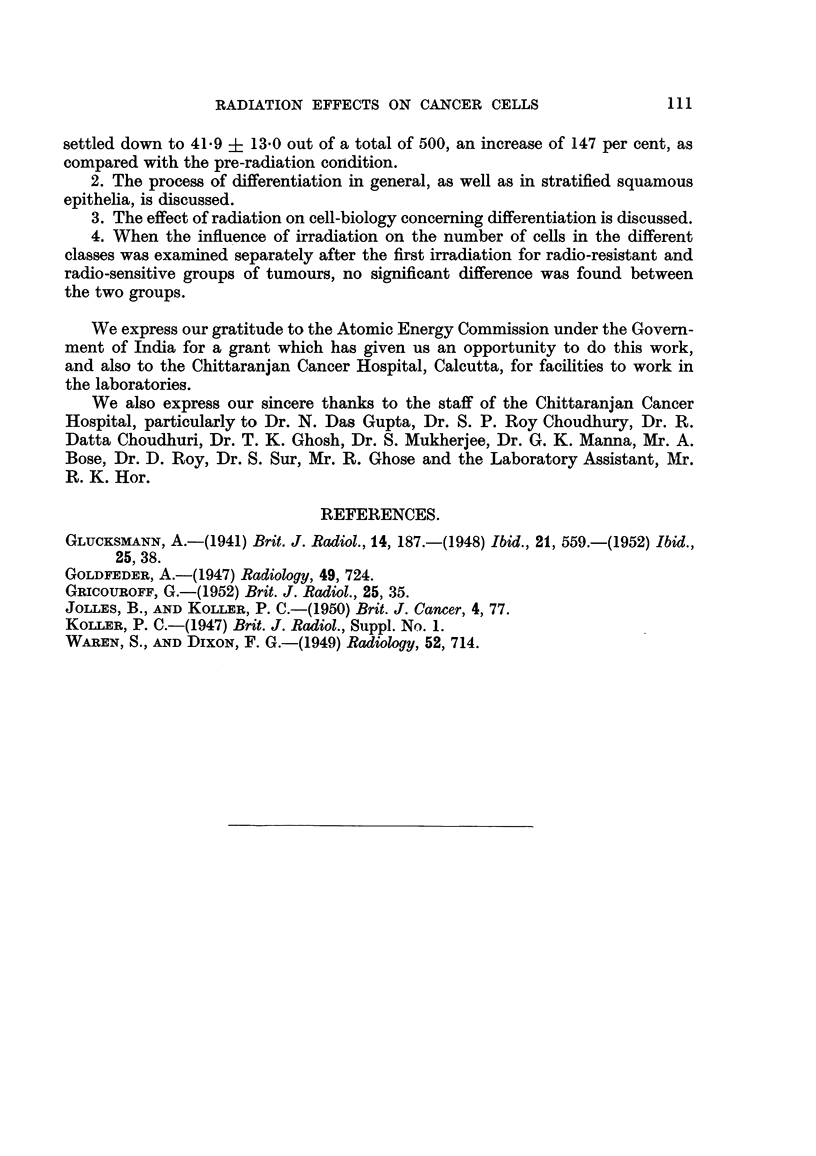# Differentiation and Radiation Effects on Cancer Cells

**DOI:** 10.1038/bjc.1954.8

**Published:** 1954-03

**Authors:** S. Mitra, P. K. De


### DIFFERENTIATION AND RADIATION EFFECTS ON CANCER CELLS.

S. MITRA AND P. K. DE.
*From the Chittaranjan Cancer Hospital, Calcutta, India.*

Received for publication January 4, 1954.

THE study of the effect of radiation on cancer cells presents problems of fundamental importance. It is still an open question in the field of radiobiology as to whether the ionising effect of radiation directly induces differentiation in cancer cells. Glucksmann (1952) observed that radiation induces differentiation in addition to mutation and degeneration. He has also shown that radio-sensitive and radio-resistant cancer cells react to radiation in a different way after a preliminary radiation.

Waren and Dixon (1949) are of opinion that radiation does not induce differentiation of cancer cells. Jolles and Koller (1950) remark that the apparent increase of differentiating cells after radiation on cancer cells, is only relative and is caused by destruction of other types of cells. Glucksmann's method of estimating the radio-sensitivity of cancer cells with preliminary radiation has been criticised by Gricouroff (1952) who is of the opinion that radio-sensitivity and differentiation of cancer cells are related to stromal nutrition.

Thus it is evident from the literature that our knowledge of the mode of action on radiation is not decisive. With this uncertain knowledge of the biological effect of radiation on cancer cells, we have made an attempt to study : (1) the problem of the induction of differentiation on cancer cells by radiation, and (2) the problem of differential radiation-response of radio-resistant and radio-sensitive cancer cells.

### METHOD AND MATERIAL

### A. *Tissue collection.*

Tissues from the growing edges of epidermoid carcinoma of the cervix were collected from 91 patients before radium application and after 7000 r, 14,000 r and 21,000 r of radium applications. For the study of the effect of first 7000 r radium, we have collected tissues 7 days after the exposure. For the study of the effect of second 7000 r radium (total 14,000 r), we have collected tissues from the same patients, just before the third application of radium, i.e. 21 days after the second 7000 r, radium exposure. Next, tissues were collected from the same patients 3 months after the third radium (total 21,000 r) application.

All these collections were made from growing tips of epidermoid carcinomatous areas of the cervix from 4 to 6 different places, by the same experienced gynaecologist (senior author), avoiding necrotic and inflammatory areas.

### B. *Fixation of tissues and preparation of sections.*

Tissues were fixed in Susa immediately after collection. All these 4 to 6 pieces of tissues were placed in the same paraffin block, and sections were made

5$\mu$ thick. Every fifteenth section from the ribbon was mounted and stained, the remainder being discarded. 60 such sections were selected (each containing 4 to 6 sections as collected from different regions of the cervix), so that for each study 240 to 360 sections at the interval of 75$\mu$ (5 $\times$ 15) were examined. All these sections were stained in haematoxylin and eosin.

*Study.*

(a) *Site selection.*—Growing tips of interpapillary downgrowths of active carcinomatous cells are selected for our study. In selecting these, we had to keep in mind the presence of neighbouring hyperplastic stratified epithelia which were carefully avoided. Our study was strictly confined to those cancerous areas which were characterised by anisocytosis, anisonucleosis, abundance of mitotic cells, loss of polarity of epithelial cells and just breakage of basement membrane. In addition to above precautions and in order to study only the actively growing carcinomatous areas, we have avoided those areas which contain cornification and cell-nest formation, necrosis and haemorrhagic areas.

In some post-radiation tissue (specially after 14,000 r and 21,000 r) there were only several scattered isolated groups of cancer cells. Here we had no selection.

(b) *Grouping of carcinoma cells.*—Carcinoma cells are grouped into four biological phases, namely, resting (inter-kinetic), mitotic, differentiating and degenerating phases (Glucksmann 1941).

(c) *Counting.*—We have made the field of our study very limited by placing a quadrilateral space in the eye-piece and thus making a grid for our study. With this eye-piece, we counted 500 carcinoma cells from the site of selection under oil-immersion lens ($\times$ 1700 magnification), classifying them as resting (inter-kinetic), mitotic, differentiating and degenerating cells.

In some post-radiation cases (specially after 14,000 r and 21,000 r) we could not find more than a total of 100 carcinoma cells. We have discarded these cases from our list. Thus we had to discard 9 cases from our 100 completed cases. The present study is based on 91 complete cases only.

### DISCUSSION.

Our problem is concerned with permanent changes in cell morphology after radiation, which includes delayed effects and recovery effects. The induction of differentiation in cancer cells is one of such delayed effects. It is for this reason, that we have collected tissues, as mentioned previously, on the 7th day after first radiation with 7000 r, on the 21st day after the second 7000 r radiation and finally 3 months after a total radiation of 21,000 r.

Before coming to our problem proper, we made a comparative study of the cellular population of the differentiating phases of the stratified squamous epithelia of the normal cervix and of epidermoid carcinoma of the cervix. Table I shows that the cell population of the differentiating phase of the normal cervix is 48·17 $\pm$ 0·41 per cent compared with 3·57 $\pm$ 0·14 per cent of the epidermoid carcinoma of the cervix.

We find the incidence of the variation of standard error in post-radiation conditions is high. This could not be avoided in spite of all precautions. It might be due to variation on the biology of cancer cells, and also to altered conditions of different patients. We have collected tissues from patients of

TABLE I.—*Percentage Distribution of Biological Phases in Normal and Carcinomatous Cervical Epithelium.*

| 1. Cervix. | 2. No. of cases. | 3. Resting phase. | 4. Mitotic phase. | 5. Differentiating phase. | 6. Degenerating phase. | 7. Preparatory phase (resting and mitotic). | 8. Cellular populations not coming to maturity (7 – 5) | 9. Retention in preparatory phase (8 – 6) |
|---|---|---|---|---|---|---|---|---|
| Stratified squamous epithelia of normal cervix | 56 | 48·78 ± 0·54 | 0·75 ± 0·05 | 48·17 ± 0·41 | 2·30 ± 0·15 | 49·53 ± 0·55 | 1·36 ± 1·10 | *Nil* |
| Epidermoid carcinoma of cervix | 439 | 84·16 ± 0·32 | 4·68 ± 0·10 | 3·57 ± 0·14 | 7·59 ± 0·25 | 89·01 ± 0·20 | 85·00 ± 0·23 | 77·84 |

different ages, different races and of different clinical stages, which might be responsible for increased variation of standard errors. Goldfeder (1947) has shown that the same morphological type of cancer cells differ in their character of radio-sensitivity. Koller (1947) observed that the mean number of chromosome fragments per cell, differed in cell samples taken from different regions of the same tumour after the same radiation treatment. He suggested that these may be due to differences of radio-sensitivity of different cell components.

In our present study we have observed that the mean of differentiating cells before radiation was 17·0 ± 1·45 and after successive radiations from 7000 r to 21,000 r, the mean of differentiating cells went up to 49·8 ± 8·30 (i.e. an increase of 194 per cent) and ultimately settled down to 41·9 ± 13·09 (i.e. an increase of 147 per cent from the pre-radiation number). It is evident from these figures that there is a definite increase of differentiating cells after radiation. Similarly there were significant changes in all other cellular populations as shown by the value of ' t ' test in Table III.

TABLE II.—*Mean Cellular Populations of Different Biological Phases, with Standard Error, before and after Radiation.*

| | No. of cases | Resting phase. | Mitotic phase. | Differentiating phase. | Degenerating phase. |
|---|---|---|---|---|---|
| Pre-radiation | 91 | 424·7 ± 1·75 | 22·8 ± 0·97 | 17·0 ± 1·45 | 35·5 ± 2·23 |
| After 1st 7000 r | 91 | 74·3 ± 7·0 | 9·0 ± 0·92 | 36·2 ± 4·01 | 380·8 ± 7·77 |
| After 2nd 7000 r | 32 | 90·3 ± 22·56 | 3·3 ± 0·87 | 49·8 ± 8·30 | 356·6 ± 23·25 |
| After 3rd 7000 r | 15 | 224·8 ± 27·84 | 8·4 ± 2·57 | 41·9 ±13·04 | 224·9 ± 30·27 |

TABLE III.—*Evaluation of Cellular Changes by "t" Test.*

| Contrast. | Mean. | S.D. | ' t.' | P 5 per cent.* | P 1 per cent.* | Significance. |
|---|---|---|---|---|---|---|
| Resting phase | 350·67 | 78·26 | 42·79 | 1·987 | 2·632 | Highly significant at both levels |
| Mitotic phase | 13·82 | 10·23 | 12·89 | 1·987 | 2·632 | Ditto |
| Differentiating phase | 18·40 | 36·58 | 4·80 | 1·987 | 2·632 | ,, |
| Degenerating phase | 345·27 | 74·41 | 44·27 | 1·987 | 2·632 | ,, |

* Taken from Statistical Tables for Biological, Agricultural and Medical Research by R. A. Fisher and F. Yates.

The trend of distribution shows also significant changes after second and third 7000 r, but the 't' test could not be applied because the number of cancer positive cases decreased materially after the 2nd and 3rd radiation.

Our second problem is concerned with the possibility of predicting radio-sensitivity of epidermoid cancer of cervix after preliminary radiation with 7000 r. It has been shown previously (Table II) that after the first radiation with 7000 r, there was a uniform reduction of cancer cell population of both resting and mitotic phases, and similarly a uniform increase of cell population of the differentiating and degenerating phases. We have not found any differential variation in the four biological phases of cancer cells after preliminary radiation to distinguish a radio-sensitive case from a radio-resistant one, as has been found by Glucksmann (1948).

In our present series of 91 cases, no cancer cells were found in the final biopsy after 21,000 r in 76 cases, which may be considered as radio-sensitive cases. Cancer cells were persistent in the remaining 15 cases under similar conditions and they may be termed radio-resistant cases.

On further analysis of our data, arranging them into final cancer-positive and cancer-negative groups after full radiation with 21,000 r, we do not find any significant variation of cell population in different phases after the preliminary radiation with 7000 r (Table IV). The difference between the cellular populations of the four biological phases of final cancer-positive and final cancer-negative cases, does not emerge into statistical significance as shown by the value of $\chi^2$ (Table V) which indicates the degree of difference between these two distributions.

TABLE IV.—*Mean Distribution of Final Cancer-positive and Final Cancer-negative Cases after first* 7000 *r Radiation.*

| Phases. | After first 7000 r radiation: Final cancer-positive cases. | After first 7000 r radiation: Final cancer-negative cases. |
|---|---|---|
| Resting | 98·47 ± 19·41 | 69·30 ± 7·67 |
| Mitotic | 12·40 ± 2·96 | 8·30 ± 0·97 |
| Differentiating | 30·07 ± 8·86 | 37·30 ± 3·73 |
| Degenerating | 359·07 ± 21·27 | 385·12 ± 27·28 |

TABLE V.

| Contrast. | Degrees of freedom. | $\chi^2$. | P 5 per cent. | P 1 per cent. |
|---|---|---|---|---|
| Pre-radiation | 3 | 5·49 | 7·815 | 11·341 |
| After first 7000 r radiation | 3 | 2·83 | 7·815 | 11·341 |

The $\chi^2$ values are not significant at both 5 per cent and 1 per cent levels.

### SUMMARY

1. Cells were classified as in the resting, mitotic, differentiating, or degenerating phase. In pre-radiation biopsies, 17·0 ± 1·4 out of a total of 500 cells were classified as differentiating. After irradiation the number in this class rose to 49·8 ± 8·3 out of a total of 500, an increase of 194 per cent; and ultimately settled down to $41 \cdot 9 \pm 13 \cdot 0$ out of a total of 500, an increase of 147 per cent, as compared with the pre-radiation condition.

2. The process of differentiation in general, as well as in stratified squamous epithelia, is discussed.

3. The effect of radiation on cell-biology concerning differentiation is discussed.

4. When the influence of irradiation on the number of cells in the different classes was examined separately after the first irradiation for radio-resistant and radio-sensitive groups of tumours, no significant difference was found between the two groups.

We express our gratitude to the Atomic Energy Commission under the Government of India for a grant which has given us an opportunity to do this work, and also to the Chittaranjan Cancer Hospital, Calcutta, for facilities to work in the laboratories.

We also express our sincere thanks to the staff of the Chittaranjan Cancer Hospital, particularly to Dr. N. Das Gupta, Dr. S. P. Roy Choudhury, Dr. R. Datta Choudhuri, Dr. T. K. Ghosh, Dr. S. Mukherjee, Dr. G. K. Manna, Mr. A. Bose, Dr. D. Roy, Dr. S. Sur, Mr. R. Ghose and the Laboratory Assistant, Mr. R. K. Hor.

### REFERENCES.

GLUCKSMANN, A.—(1941) *Brit. J. Radiol.*, **14**, 187.—(1948) *Ibid.*, **21**, 559.—(1952) *Ibid.*, **25**, 38.
GOLDFEDER, A.—(1947) *Radiology*, **49**, 724.
GRICOUROFF, G.—(1952) *Brit. J. Radiol.*, **25**, 35.
JOLLES, B., AND KOLLER, P. C.—(1950) *Brit. J. Cancer*, **4**, 77.
KOLLER, P. C.—(1947) *Brit. J. Radiol.*, Suppl. No. 1.
WAREN, S., AND DIXON, F. G.—(1949) *Radiology*, **52**, 714.